# Implementing sleep apnea care to improve positive airway pressure adherence here and across the pond

**DOI:** 10.1093/ajrccm/aamag248

**Published:** 2026-05-21

**Authors:** Samantha Amanda Sathyapala, Sairam Parthasarathy

**Affiliations:** National Heart and Lung Institute, Imperial College London, London, United Kingdom; National Heart and Lung Foundation, London, United Kingdom; Guy’s and St Thomas’ NHS Foundation Trust, London, United Kingdom; Division of Pulmonary, Allergy, Critical Care, and Sleep Medicine, University of Arizona, Tucson, AZ, United States

Poor adherence to continuous positive airway pressure (CPAP) hampers benefits to patients with obstructive sleep apnea (OSA),^1^ and adherence rates have remained poor for 30 years.^2^^,^^3^ Given the harm and cost resulting from poor CPAP adherence,^4^ this needs addressing urgently. More than 40 interventions to improve patient adherence to CPAP for OSA have been developed,^5^ but none have been adopted by health care systems. Here, we take a novel and systematic approach to identifying the major barriers to implementation of these interventions and potential solutions going forward on both sides of the pond. We use the PRISM (Pragmatic Robust Implementation and Sustainability Model)/RE-AIM (Reach, Effectiveness, Adoption, Implementation, and Maintenance) implementation science framework. This framework was developed to improve the adoption and sustainable implementation of evidence-based interventions, programs, and policies into various settings.^6^ We followed the approach that the PRISM authors have taken when identifying barriers to implementation of other interventions.^7^ This involved focusing on the PRISM model, which describes contextual factors mediating relationships between implementation strategies and implementation outcomes that influence intervention implementation, to deduce key barriers. From these, solutions are left to be inferred without further use of the framework (see Figure 1 for full analysis). From this, we conclude that the main barrier to CPAP adherence intervention implementation has been intervention design and development without consideration of feasibility. We propose that digital or non-sleep staff-implemented CPAP adherence interventions are potential solutions.

**Figure 1:**
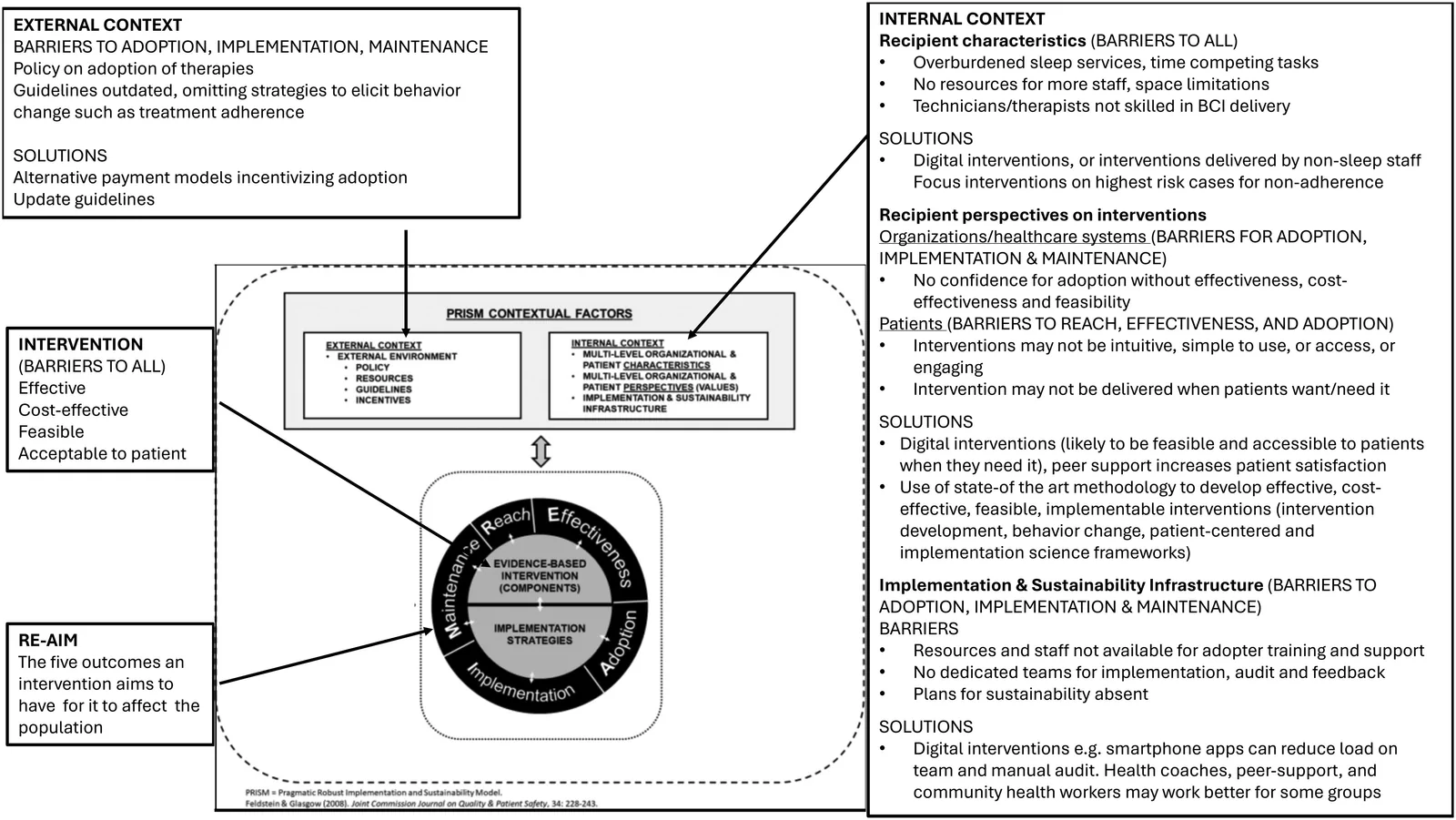
Barriers and solutions to implementation of interventions, programs, or policies that can improve patient adherence to CPAP for OSA using the PRISM framework. Schematic illustrating the PRISM domains and RE-AIM outcomes and highlighting specific barriers and potential solutions to implementation of interventions/policies/programs to improve CPAP adherence. Abbreviations: A, adoption; CPAP, continuous positive airway pressure; E, effectiveness/efficacy; I, implementation; M, maintenance; OSA, obstructive sleep apnea; PRISM, Pragmatic Robust Implementation and Sustainability Model; R, reach; RE-AIM, Reach, Effectiveness, Adoption, Implementation and Maintenance.

Key to implementation success in the PRISM/RE-AIM framework is intervention design, as implementation depends on the fit between intervention and the system in which it is implemented, and the system can rarely be changed. The intervention therefore needs to be not only effective and cost-effective (as assessed by cost-utility and cost-benefit analyses) but also feasible. Feasibility is defined as “the extent to which a new treatment or innovation can be successfully used or carried out within a given agency or setting. This is related to appropriateness but may include other concerns specific to an agency or organization like resources or staff training needs.”^8^ Appropriateness has often been defined as “the extent to which the expected health benefits of a procedure exceed the expected negative consequences by a sufficiently wide margin that the procedure is worth doing.”^9^ Over time, appropriateness has become more directly associated with cost.^9^ Poor feasibility and lack of cost-effectiveness data have previously been suggested as key hurdles for adoption of current CPAP adherence interventions.^10^

## What does the PRISM/RE-AIM framework tell us about the issues with existing interventions?

### Internal context

The internal context comprises the organizations/health care systems/staff, patients’ characteristics and perspectives to the intervention, and any infrastructure preventing intervention implementation (see Figure 1). Staff shortages, and inadequate resources, for example to increase staff numbers, are a critical implementation barrier to interventions in studies such as Chapman et al.^11^ Since competitive bidding, there has been a significant reduction in durable medical equipment (DME) businesses (hence, DME staff) in many areas of the United States.^12^ Similarly, understaffing of the UK National Health Service sleep services was reported in 2014,^13^ and the situation has not improved since. Obstructive sleep apnea patients feel UK sleep staff are too busy to support them with using CPAP (unpublished survey). The European Union countries are experiencing a similar health care worker crisis.^14^ In this context, several staff-intensive motivational enhancement therapy (MET) interventions have been developed and continue to be adapted for CPAP adherence.^5^^,^^15^ Motivational enhancement therapy is based on motivational interviewing (MI)—a person-centered style of counseling.^16^ In the two most recent studies, MET was delivered 1:1 by a nurse practitioner trained by a psychologist with MI expertise for 30-45 minutes on four occasions,^17^ and in the other, delivered for at least 45 minutes, six times for those with poor adherence.^18^ The huge staff burden imposed, among other issues such as trainer availability, sleep staff uncertainty about willingness to do work outside their primary role,^11^ and the minimal effectiveness of MET from meta-analysis^15^ (which questions appropriateness), render this intervention type unfeasible.

Interventions developed for delivery by smartphone apps, other digital platforms, or telemedicine approaches and chatbots using artificial intelligence circumvent the need for staff.^10^^,^^19^ Digital interventions are likely to be more cost-effective than face-to-face interventions, as their set-up costs and minimal running costs are generally lower than staff salary costs. Behavior change, including medication adherence,^20^ increased physical activity, smoking, and alcohol cessation or reduction, has been elicited with digital tools,^21^ although many apps have been low quality with few behavior change techniques^20^^,^^21^ These limitations are true of two of the smartphone apps developed to improve CPAP adherence.^22^^,^^23^ The third app had a good signal of efficacy in a randomized controlled feasibility study of 100 patients but increased staff calls (about the app) more than twice the usual care group, increasing staff ­burden.^24^ These limitations are, however, potentially remediable.

Community dwelling persons, such as peers with OSA, can also implement interventions instead of staff. Peer interventions have been effective for inducing dietary behavior change, for ­example.^25^ Our peer mentoring intervention (support from a trained peer using CPAP), published in the *American Journal of Respiratory and Critical Care Medicine* last year, has demonstrable effectiveness.^26^ Cost-effectiveness data are being acquired. Digital peer support platforms could be a lower cost, more feasible alternative. These are in early development for other conditions^27^ and have yet to be developed for CPAP adherence.

### External context

Lower cost, digital interventions that are simple to integrate into health care pathways are attractive to buyers, especially when health care systems are stretched,^14^^,^^28^ as they are highly feasible.

In conclusion, CPAP therapy is likely to remain the most common and first-line therapy for most OSA patients, so under­standing curtailment to implementation of CPAP adherence interventions is crucial. Using a systematic implementation science approach to identify barriers, we propose that failure to consider feasibility in the intervention design has been the fundamental flaw. Designing digital and possibly peer CPAP adherence interventions are potential solutions and should be the direction going forward.

## Supplementary Material

aamag248_Supplementary_Data

## Appendix

**PRISM DOMAINS** 

**Internal Context** 

***Recipients*** 

*Characteristics* (eg, settings, staff, patients)

Organizational: those that affect organizations’ ability to successfully change behaviors in a clinical area (eg, clinical leadership, management support and communication)

Patient (eg, demographics, health beliefs, and health literacy)

*Perspectives of the intervention*

Organizational and staff: characteristics such as organizational readiness for the intervention, the intervention’s perceived evidence strength, and compatibility with existing workflow

Patient (recipients): characteristics such as perceptions of the intervention’s patient-centeredness, patient barriers and service and access, and estimated impact and ease of use

***Implementation and Sustainability Infrastructure*** 

These include adopter training and support, dedicated teams for intervention implementation, ongoing audit and feedback of the intervention, and resources and plans for maintenance of effects of intervention.

**External Context** 

***External Environment*** 

These include characteristics such as relevant policies, market forces, regulatory environment, and community resources.

**RE-AIM OUTCOMES** 

*Reach*: the absolute number, proportion, and representativeness of individuals who are willing to participate in a given intervention

*Effectiveness (or Efficacy)*: the impact of an intervention on important outcomes

*Adoption*: the absolute number, proportion, and representativeness of settings and intervention agents (people who deliver the program) who are willing to initiate a program

*Implementation*: refers both to the intervention agents’ fidelity to the intervention’s protocol and the clients’ use of the intervention strategies

*Maintenance*: the extent to which a program or policy becomes part of the routine organizational practices and policies and has long-term effects on individuals after the intervention has ceased

## References

[1] Bocoum AM , Bailly S, Joyeux-Faure M, et al Long-term outcomes of CPAP-treated sleep apnea patients: impact of blood-pressure responses after CPAP initiation and of treatment adherence. Sleep Med. 2023;109:25-31. 10.1016/j.sleep.2023.06.022 37399713

[2] Rotenberg BW , Murariu D, Pang KP. Trends in CPAP adherence over twenty years of data collection: a flattened curve. J Otolaryngol Head Neck Surg. 2016;45:43-50. 10.1186/s40463-016-0156-0 27542595 PMC4992257

[3] Dielesen J , Ledwaba-Chapman LJ, Kasetti P, et al Six early CPAP-usage behavioural patterns determine peak CPAP adherence and permit tailored intervention, in patients with obstructive sleep apnoea. Thorax. 2025;80:300-308. 10.1136/thorax-2024-221763 40015971 PMC12015089

[4] Sánchez-de-la-Torre M , Gracia-Lavedan E, Benitez ID, et al Adherence to CPAP treatment and the risk of recurrent cardiovascular events: a meta-analysis. JAMA. 2023;330:1255-1265. 10.1001/jama.2023.17465 37787793 PMC10548300

[5] Askland K , Wright L, Wozniak DR, Emmanuel T, Caston J, Smith I. Educational, supportive and behavioural interventions to improve usage of continuous positive airway pressure machines in adults with obstructive sleep apnoea. Cochrane Database Syst Rev. 2020;4:CD007736. 10.1002/14651858.CD007736.pub3 32255210 PMC7137251

[6] Glasgow RE , Harden SM, Gaglio B, et al RE-AIM planning and evaluation framework: adapting to new science and practice with a 20-year review. Front Public Health. 2019;7:64. 10.3389/fpubh.2019.00064 30984733 PMC6450067

[7] Matlock DD , Fukunaga MI, Tan A, et al Enhancing success of Medicare’s shared decision making mandates using implementation science: examples applying the Pragmatic Robust Implementation and Sustainability Model (PRISM). MDM Policy Pract. 2020;5:1-13. 10.1177/2381468320963070 PMC7570787 33117890

[8] Rabin BSK , Glasgow R, Ford B, et al Dissemination and implementation models in health 2014-2026. ACCORDS Dissemination and Implementation Science Program. Accessed April 13, 2026. https://dissemination-implementation.org/constructs/acceptability-feasibility/

[9] Sanmartin C , Murphy K, Choptain N, et al Appropriateness of healthcare interventions: concepts and scoping of the published literature. Int J Technol Assess Health Care. 2008;24:342-349. 10.1017/S0266462308080458 18601803

[10] Bakker JP , Weaver TE, Parthasarathy S, Aloia MS. Adherence to CPAP: what should we be aiming for, and how can we get there? Chest. 2019;155:1272-1287. 10.1016/j.chest.2019.01.012 30684472

[11] Chapman A , Buccheri A, Mohotti D, et al Staff-reported barriers and facilitators to the implementation of healthcare interventions within regional and rural areas: a rapid review. BMC Health Serv Res. 2025;25:331. 10.1186/s12913-025-12480-8 40033247 PMC11877690

[12] Centers for Medicare & Medicaid Services. Public Comments State Plan Amendment (SPA) 17-0015 Durable Medical Equipment (DME) and Medical Supply Reimbursement 2017. Accessed January 4, 2026. https://medicaid.ms.gov/wp-content/uploads/2017/11/MS-SPA-17-0015-Public-Comments.pdf#:∼:text=If%20MS%20DOM%20is%20not%20allowed%20to,to%20beneficiaries%20at%20rates%20below%20their%20cost

[13] British Lung Foundation. OSA: the experts’ viewpoint. 2014. Accessed January 4, 2026. https://www.asthmaandlung.org.uk/sites/default/files/BLF-OSA-conference-February-2014.pdf

[14] Kuhlmann E. The health workforce crisis in the European Union European Parliament. 2025. Accessed March 15, 2026. https://www.europarl.europa.eu/RegData/etudes/BRIE/2025/772481/ECTI_BRI(2025)772481_EN.pdf

[15] Chaiard J , Bhatarasakoon P. Effectiveness of behavioral and psychosocial interventions for continuous positive airway pressure adherence among patients with obstructive sleep apnea: a systematic review and meta-analysis. Appl Nurs Res. 2023;69:151654. 10.1016/j.apnr.2022.151654 36635010

[16] Rollnick S , Miller WR. What is motivational interviewing? Behav Cogn Psychother. 1995;23:325-334. 10.1017/S135246580001643X 19364414

[17] Miller JN , Kupzyk K, Zheng C, et al Nurse practitioner-led, virtually delivered, motivational enhancement and device support intervention to improve CPAP adherence: a feasibility randomized control trial. Heart Lung J Crit Care. 2024;63:119-127. 10.1016/j.hrtlng.2023.10.005 37879189

[18] Rapelli G , Pietrabissa G, Angeli L, et al Study protocol of a randomized controlled trial of motivational interviewing-based intervention to improve adherence to continuous positive airway pressure in patients with obstructive sleep apnea syndrome: the MotivAir study. Front Psychol. 2022;13:947296. 10.3389/fpsyg.2022.947296 36092104 PMC9456614

[19] Singh B , Olds T, Brinsley J, et al Systematic review and meta-analysis of the effectiveness of chatbots on lifestyle behaviours. NPJ Digit Med. 2023;6:118. 10.1038/s41746-023-00856-1 37353578 PMC10290125

[20] Polsook R , Supametaporn P, Suktrakul S, Wuttisittikulkij L. Self-management technology of medication adherence in patient chronic disease: a systematic review and meta-analysis. Worldviews Evid Based Nurs. 2026;23:e70137. 10.1111/wvn.70137 41930416

[21] McKay FH , Wright A, Shill J, Stephens H, Uccellini M. Using health and well-being apps for behavior change: a systematic search and rating of apps. JMIR Mhealth Uhealth. 2019;7:e11926. 10.2196/11926 31274112 PMC6637726

[22] Petrov ME , Epstein DR, Krahn L, et al SleepWell24, a smartphone application to promote adherence to positive airway pressure therapy: feasibility and acceptability in a randomized controlled trial. Behav Sleep Med. 2024;22:420-432. 10.1080/15402002.2023.2289442 38032115 PMC11136882

[23] Pfammatter AF , Hughes BO, Tucker B, Whitmore H, Spring B, Tasali E. The development of a novel mHealth tool for obstructive sleep apnea: tracking continuous positive airway pressure adherence as a percentage of time in bed. J Med Internet Res. 2022;24:e39489. 10.2196/39489 36469406 PMC9764150

[24] Franke C , Piezonna F, Schäfer R, Grimm A, Loris LM, Schwaibold M. Effect of a digital patient motivation and support tool on CPAP/APAP adherence and daytime sleepiness: a randomized controlled trial. Sleep Biol Rhythms. 2024;22:49-63. 10.1007/s41105-023-00479-9 38469583 PMC10899947

[25] Moore SE , McMullan M, McEvoy CT, McKinley MC, Woodside JV. The effectiveness of peer-supported interventions for encouraging dietary behaviour change in adults: a systematic review. Public Health Nutr. 2019;22:624-644. 10.1017/S1368980018003294 30501679 PMC6411137

[26] Parthasarathy S , Wendel C, Grandner MA, et al Peer-driven intervention for care coordination and adherence promotion for obstructive sleep apnea: a randomized, parallel-group clinical trial. Am J Respir Crit Care Med. 2025;211:248-257. 10.1164/rccm.202309-1594OC 39441133 PMC12392785

[27] Yeo G , Fortuna KL, Lansford JE, Rudolph KD. The effects of digital peer support interventions on physical and mental health: a review and meta-analysis. Epidemiol Psychiatr Sci. 2025;34:e9. 10.1017/S2045796024000854 39945388 PMC11886969

[28] Rasanathan K. How can health systems under stress achieve universal health coverage and health equity? Int J Equity Health. 2024;23:244-248. 10.1186/s12939-024-02293-2 39574087 PMC11580394

